# Supplementary material for: Multilevel correlates of abdominal obesity in adolescents and youth living with HIV in peri-urban Cape Town, South Africa
Source: PLoS One. 2023 Jan 24;18(1):e0266637. doi: 10.1371/journal.pone.0266637 (PMC9873196; doi:10.1371/journal.pone.0266637)
Supplement: S1 Table — (DOCX) [file pone.0266637.s001.docx]

S1 Table: Definition and measurement of derived variables

| **Variable** | **Data or Indicator Required** | | **Categories or Composition** |
| --- | --- | --- | --- |
| **INDIVIDUAL CHARACTERISTICS** | | | |
| Demographic characteristics | Age, sex, ever pregnant and parity | | Age categories: 15–17, 18–19, 20–21 and 22–24 years.  Parity: 0 children, 1 child or more |
| School attendance and performance | Educational attainment, current occupation, absenteeism, ever repeated a grade at school | | Education: primary, some secondary, matric or equivalent, some tertiary.  Occupation: employed, in school /college/ university/other tertiary,  Neither in education/ employment nor training  Absenteeism: 0 days, 1 or more days |
| Socioeconomics | Youth Multidimensional Poverty Index (YMPI)^^[[1]](#footnote-1)^^ Five dimensions and 11 indicators [1]. | | General health and functioning, education, living standards, asset deprivation, economic opportunities. |
| **Cardio-metabolic risks** | | | |
| Anthropometric measurements | Weight, height, waist circumference^^[[2]](#footnote-2)^^,  hip circumference. | |  |
| Hypertension/ elevated blood pressure | Measured blood pressure (systolic / diastolic) | | Normal BP: <130/85 mmHg,  Elevated BP: SBP 130-139, DBP 85-89 mmHg,  Hypertension: >140/90 mmHg. |
| Family history | Parental medical history of diabetes/ stroke/ high blood pressure | |  |
| **Knowledge and behaviour** | | | |
| Dietary intake and nutrition | Frequency of consumption of fruit, vegetables, wholegrains, fast-foods, deep-fried foods, cakes and biscuits, sugar-sweetened beverages [2]. | | Weekly portions consumed.  Frequency: Never, once a week,  2 - 4 times a week,  frequently (5-6 times a week),  Daily or more than once a day. |
| Physical activity and sedentary behaviour | Questions from IPAQ-short form [3]: Metabolic Equivalents and classification into insufficient, moderate or high levels of physical activity [4]. | | Insufficient PA: <600 MET/ week  High PA: >3000 METs/week.  Sedentary behaviour: 3 or more hours sedentary in a typical day. |
| Nutrition Knowledge | Overall GNKQ Score:  Four sub-domains according to revised GNKQ [5]. | | 1. Dietary recommendations.  2. Food Groups.  3. Healthy Food choices.  4. Diet-disease and weight management. |
| **HOME ENVIRONMENT** | | | |
| **Dwelling characteristics** | | | |
| Housing | Type of dwelling.  History of flooding/fire.  Access to amenities.  Sanitation.  Main source of water.  Household waste/refuse removal.  Thermal comfort. | | Formal or informal dwelling.  Fuel used for lighting, heating and cooking.  Flush toilet inside house or yard.  Piped water inside dwelling, piped water on site or yard, public tap.  Waste removal: removed weekly, removed or less than weekly, communal dump, own dump, other.  Any discomfort experienced in summer, spring or autumn and winter. |
| Food security | Household Food Insecurity Access Scale (HFIAS) [6] | | HFIAS prevalence categories:  Food secure, mildly food insecure access, moderately food insecure access, severely food insecure access. |
| Primary caregiver relationship | Orphanhood status: Parents deceased or alive.  Primary caregiver | | Primary caregiver relationship: Biological parent as a primary caregiver. |
| Household composition | Family structure (whom they live with).  Number of people in the household who live together regularly.  Number of children (0-14 years old) and older persons (≥ 65 years); Working-age adults (15-64 years old). | | Whom adolescent lives with: Biological parent, grandparent, siblings, other extended family member, non-family member (social worker/ care worker, foster carer). |
| **COMMUNITY LEVEL FACTORS** | | | |
| Experiences of stigma | HIV Stigma Scale for Adolescents Living with HIV (ALHIV-SS) [7]. | | Anticipated stigma, internalized stigma, enacted stigma. |
| Perceptions of social capital | **Neighbourhood Social Capital** [8, 9]**:**  **(**4 items)  *‘Do you feel people trust each other in your neighbourhood (neighbourhood trust)?’’*  *‘‘Do people help each other in your neighbourhood (neighbourhood reciprocity)?”*  *‘‘People in my neighbourhood are friendly (neighbourhood friendliness)?”*  *“Do you feel like you belong in your neighbourhood (neighbourhood belonging)?”* | | We created a dichotomous variable for each response (high: strongly agree, agree and ‘neither agree or disagree’; low: ‘disagree’ and ‘strongly disagree’)  High or low neighbourhood trust  High or low neighbourhood reciprocity  High or low neighbourhood friendliness  High or low neighbourhood belonging |
| Safety & violence | 8 Sub-items from the Survey of Exposure to Community Violence scale [10];  Crime safety items from NEWS-Y | | No violence (Score < 2),  Moderate level (Score 2 – 3),  and high level of violence (4 – 8).  Mean of 6 items. |
| **BUILT ENVIRONMENT** | | | |
| Perceived Neighbourhood Environment Walkability | **Neighbourhood Environment Walkability Scale for Youth (NEWS-Y) [11]** | | |
|  | A. *Land-use mix – diversity* (“Stores, facilities in neighbourhood”) | Responses : 1-5 min (1), 6-10 min (2), 11-20 min (3), 21-30 min (4), 31+ min (5), don’t know (5)^[[3]](#footnote-3)^.  Score = Mean of 20 items. | |
|  | B. *Access to Neighbourhood recreation facilities*. | Responses like A.  Score = Mean of 14 items. | |
|  | C. *Residential density* (“Types of homes in neighbourhood”) | None (1), A few (2), Some (3), Most (4), All (5).  Score on subscale C = C1 + (12*C2) + (2*C3) + (25 *C4) | |
|  | D. *Land-use mix – access*  (“Access to services”) | *Responses:* Strongly disagree (1), Somewhat disagree (2), Somewhat agree (3),  Strongly agree (4)  Score = Mean of 6 items. | |
|  | E. *Street connectivity*  (“Streets in my neighbourhood”) | Responses like D.  Score = Mean of 3 items. | |
|  | F*. Walking/cycling facilities*  (“Places for walking and cycling”) | Responses like D.  Score = Mean of 3 items. | |
|  | G. *Neighbourhood Aesthetics* (“Neighbourhood surroundings”) | Responses like D.  Score = Mean of 4 items. | |
|  | H. *Pedestrian and automobile traffic safety* (“Safety from traffic”) | Responses like D.  Score = Mean of 7 items. | |
|  | *I. Crime safety*  (“Safety from crime”) | Responses like D.  Score = Mean of 6 items. | |
| **Transportation** | Means of transport from home to school or work every day and time taken for each. | Whether or not active transport (walking/ cycling) is part of commute. | |
| **FOOD ENVIRONMENT** | | | |
| Access to the following food places or services | Convenience or small grocery store | Walking distance defined as stores or facilities within a 5, 10, or 20-minute walk from home [11]. | |
|  | Supermarket |  |  |
|  | Fruit and vegetable market |  |  |
|  | Fast food restaurant |  |  |
|  | Non-fast-food restaurant |  |  |
|  | Coffee shop |  |  |

**References:**

1. Frame E, De Lannoy A, Leibbrandt M. Measuring multidimensional poverty among youth in South Africa at the sub-national level. 2016.

2. Katzmarzyk PT, Barreira TV, Broyles ST, Champagne CM, Chaput J-P, Fogelholm M, et al. The international study of childhood obesity, lifestyle and the environment (ISCOLE): design and methods. BMC public health. 2013;13(1):900.

3. Craig C, Marshall A, Sjostrom M, Bauman A, Lee P, Macfarlane D, et al. International Physical Activity Questionnaire-Short Form. 2017.

4. Committee IR. Guidelines for the Data Processing and Analysis of the International Physical Activity Questionnaire. 2005. 2016.

5. Kliemann N, Wardle J, Johnson F, Croker H. Reliability and validity of a revised version of the General Nutrition Knowledge Questionnaire. European journal of clinical nutrition. 2016;70(10):1174-80.

6. Coates J, Swindale A, Bilinsky P. Household Food Insecurity Access Scale (HFIAS) for measurement of food access: indicator guide: version 3. 2007.

7. Pantelic M, Boyes M, Cluver L, Thabeng M. ‘They Say HIV is a Punishment from God or from Ancestors’: Cross-Cultural Adaptation and Psychometric Assessment of an HIV Stigma Scale for South African Adolescents Living with HIV (ALHIV-SS). Child Indicators Research. 2016:1-17.

8. Novak D, Suzuki E, Kawachi I. Are family, neighbourhood and school social capital associated with higher self-rated health among Croatian high school students? A population-based study. BMJ Open. 2015;5(6):e007184-e. doi: 10.1136/bmjopen-2014-007184.

9. Furuta M, Ekuni D, Takao S, Suzuki E, Morita M, Kawachi I. Social capital and self-rated oral health among young people. Community Dentistry and Oral Epidemiology. 2012;40(2):97-104. doi: 10.1111/j.1600-0528.2011.00642.x.

10. Martinez P, Richters JE. The NIMH community violence project: II. Children’s distress symptoms associated with violence exposure. Psychiatry. 1993;56(1):22-35.

11. Rosenberg D, Ding D, Sallis JF, Kerr J, Norman GJ, Durant N, et al. Neighborhood Environment Walkability Scale for Youth (NEWS-Y): Reliability and relationship with physical activity. Preventive Medicine. 2009;49(2-3):213-8. doi: 10.1016/j.ypmed.2009.07.011.

1. Similarly derived as the South African Multidimensional Poverty Index (SAMPI) but uses the young person as the unit of analysis as opposed to the household. [↑](#footnote-ref-1)
2. Waist circumference improves the ability of BMI to identify hypertension in obese children. [↑](#footnote-ref-2)
3. Note: A ‘don’t know’ response is coded as a “5” because if it is not known whether the facility is within walking distance, the actual walk is likely more than 31 minutes. [↑](#footnote-ref-3)
