# Supplementary figures and images for: Multilevel correlates of abdominal obesity in adolescents and youth living with HIV in peri-urban Cape Town, South Africa
Source: PLoS One. 2023 Jan 24;18(1):e0266637. doi: 10.1371/journal.pone.0266637 (PMC9873196; doi:10.1371/journal.pone.0266637)

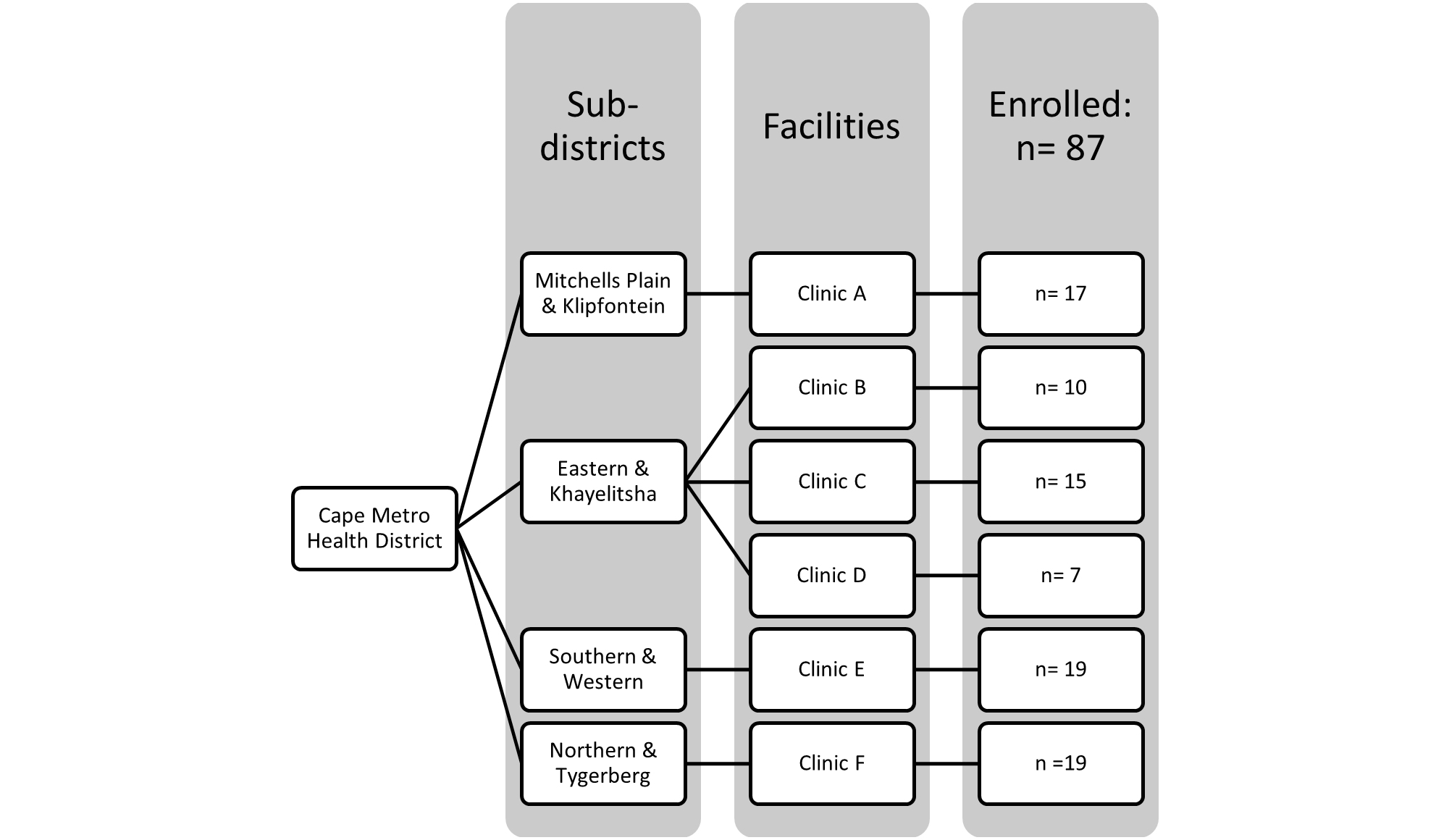

Supplement: S1 Fig — (TIF) [file pone.0266637.s002.tif]
