# Supplementary material for: Multilevel correlates of abdominal obesity in adolescents and youth living with HIV in peri-urban Cape Town, South Africa
Source: PLoS One. 2023 Jan 24;18(1):e0266637. doi: 10.1371/journal.pone.0266637 (PMC9873196; doi:10.1371/journal.pone.0266637)
Supplement: Attachment — Submitted filename: Response To Reviewers.docx [file pone.0266637.s003.docx]

21 October 2022

To: The Division Editor

Public Health and Medicine

***PLOS ONE***

Dear Editor

Thank you for giving us the opportunity to submit a revised draft of our manuscript titled “**Multilevel correlates of abdominal obesity in adolescents and youth living with HIV in peri-urban Cape Town, South Africa**” for consideration by *PLOS ONE.* We appreciate the time and effort that the editor and the reviewers have dedicated to providing feedback on our manuscript. We have been able to incorporate changes to reflect the suggestions provided by the reviewers and welcome further constructive comments if any.

Below we provide the point-by-point responses to the reviewers' comments and suggestions. All modifications in the manuscript have been highlighted as track changes. We look forward to hearing from you in due time regarding our submission and to respond to any further questions and comments you may have.

Sincerely

**Dr. Monika Kamkuemah**

Postdoctoral Fellow

Innovation Africa and Department of Architecture

Faculty of Engineering, Built Environment and Information Technology

**UNIVERSITY OF PRETORIA**

**email: monika@urbanbetter.science**

**Response to Additional Journal Requirements**

***Comment 1.*** *Please ensure that your manuscript meets PLOS ONE's style requirements, including those for file naming. The PLOS ONE style templates can be found at*

[*https://journals.plos.org/plosone/s/file?id=wjVg/PLOSOne_formatting_sample_main_body.pdf*](https://journals.plos.org/plosone/s/file?id=wjVg/PLOSOne_formatting_sample_main_body.pdf)

*and*

[*https://journals.plos.org/plosone/s/file?id=ba62/PLOSOne_formatting_sample_title_authors_affiliations.pdf*](https://journals.plos.org/plosone/s/file?id=ba62/PLOSOne_formatting_sample_title_authors_affiliations.pdf)*.*

**Response:** The manuscript has now been formatted to meet PLOS ONE's style requirements.

***Comment 2.*** *We note that you have included the phrase “data not shown” in your manuscript. Unfortunately, this does not meet our data sharing requirements. PLOS does not permit references to inaccessible data. We require that authors provide all relevant data within the paper, Supporting Information files, or in an acceptable, public repository. Please add a citation to support this phrase or upload the data that corresponds with these findings to a stable repository (such as Figshare or Dryad) and provide and URLs, DOIs, or accession numbers that may be used to access these data. Or, if the data are not a core part of the research being presented in your study, we ask that you remove the phrase that refers to these data.*

**Response:** Thank you for pointing this out. All data are accessible on Figshare at this URL: <https://figshare.com/articles/dataset/HIV_NCD_Abdominal_Obesity_Analysis_WHtR_dta/19204929>

We have removed the phrase “data not shown” from the manuscript.

***Comment 3.*** *Please review your reference list to ensure that it is complete and correct. If you have cited papers that have been retracted, please include the rationale for doing so in the manuscript text, or remove these references and replace them with relevant current references. Any changes to the reference list should be mentioned in the rebuttal letter that accompanies your revised manuscript. If you need to cite a retracted article, indicate the article’s retracted status in the References list and also include a citation and full reference for the retraction notice.*

**Response:** We have reviewed and updated the reference list. It does not contain any retracted articles.

**Response to Reviewer 1**

Reviewer's Responses to Questions

**Comments to the Author**

1. Is the manuscript technically sound, and do the data support the conclusions?

The manuscript must describe a technically sound piece of scientific research with data that supports the conclusions. Experiments must have been conducted rigorously, with appropriate controls, replication, and sample sizes. The conclusions must be drawn appropriately based on the data presented.

Reviewer #1: Yes

2. Has the statistical analysis been performed appropriately and rigorously?

Reviewer #1: Yes

3. Have the authors made all data underlying the findings in their manuscript fully available?

The [PLOS Data policy](http://www.plosone.org/static/policies.action#sharing) requires authors to make all data underlying the findings described in their manuscript fully available without restriction, with rare exception (please refer to the Data Availability Statement in the manuscript PDF file). The data should be provided as part of the manuscript or its supporting information, or deposited to a public repository. For example, in addition to summary statistics, the data points behind means, medians and variance measures should be available. If there are restrictions on publicly sharing data—e.g. participant privacy or use of data from a third party—those must be specified.

Reviewer #1: Yes

4. Is the manuscript presented in an intelligible fashion and written in standard English?

PLOS ONE does not copyedit accepted manuscripts, so the language in submitted articles must be clear, correct, and unambiguous. Any typographical or grammatical errors should be corrected at revision, so please note any specific errors here.

Reviewer #1: Yes

**5. Review Comments to the Author**

Please use the space provided to explain your answers to the questions above. You may also include additional comments for the author, including concerns about dual publication, research ethics, or publication ethics. (Please upload your review as an attachment if it exceeds 20,000 characters)

*Reviewer #1: The paper is well written.

The authors conducted a cross sectional study to investigate the prevalence of abdominal obesity and associated multilevel factors in AYLHIV in peri-urban Cape Town, South Africa. The methods are clear, results presented well, the discussion is sound, and there is coherence up to the conclusion.

The authors can just add the test they have used to check the distribution of variables under statistical analysis, which informed use of both non-parametric and parametric tests.*

Excellent!

**Response:** Thank you very much for this positive review. In response to the statistical tests used to check the distribution of variables, we have added the following to the methods section: “We used visual displays, histograms, box plots and the Shapiro–Wilk test to test for normality of the continuous variables.”

6. PLOS authors have the option to publish the peer review history of their article ([what does this mean?](https://journals.plos.org/plosone/s/editorial-and-peer-review-process#loc-peer-review-history)). If published, this will include your full peer review and any attached files.


If you choose “no”, your identity will remain anonymous but your review may still be made public.


**Do you want your identity to be public for this peer review?** For information about this choice, including consent withdrawal, please see our [Privacy Policy](https://www.plos.org/privacy-policy).

Reviewer #1: No.
