# Supplementary material for: Multilevel correlates of abdominal obesity in adolescents and youth living with HIV in peri-urban Cape Town, South Africa
Source: PLoS One. 2023 Jan 24;18(1):e0266637. doi: 10.1371/journal.pone.0266637 (PMC9873196; doi:10.1371/journal.pone.0266637)
Supplement: Attachment — Submitted filename: Response To Reviewers.docx [file pone.0266637.s004.docx]

07 January 2023

To: Professor Blake Byron Walker

Academic Editor

**PLOS ONE**

Dear Editor

Thank you for giving us the opportunity to re-submit a revised draft of our manuscript titled “**Multilevel correlates of abdominal obesity in adolescents and youth living with HIV in peri-urban Cape Town, South Africa**” for consideration by *PLOS ONE.* We appreciate the time and effort that the editor and the reviewers have dedicated to providing feedback on our manuscript. We have been able to incorporate changes to reflect the suggestions provided by the reviewers and welcome further constructive comments if any.

Below we provide the point-by-point responses to the reviewers' comments and suggestions. All modifications in the manuscript have been highlighted as track changes. We look forward to hearing from you in due time regarding our submission and to respond to any further questions and comments you may have.

Sincerely

**Dr Monika Kamkuemah**

Postdoctoral Fellow

Innovation Africa and Department of Architecture

Faculty of Engineering, Built Environment and Information Technology

**UNIVERSITY OF PRETORIA**

**email: monika@urbanbetter.science**

**Response to Reviewers’ comments**

Reviewer's Responses to Questions

**Comments to the Author**

1. If the authors have adequately addressed your comments raised in a previous round of review and you feel that this manuscript is now acceptable for publication, you may indicate that here to bypass the “Comments to the Author” section, enter your conflict of interest statement in the “Confidential to Editor” section, and submit your "Accept" recommendation.

Reviewer #1: All comments have been addressed

Reviewer #2: (No Response)

2. Is the manuscript technically sound, and do the data support the conclusions?

The manuscript must describe a technically sound piece of scientific research with data that supports the conclusions. Experiments must have been conducted rigorously, with appropriate controls, replication, and sample sizes. The conclusions must be drawn appropriately based on the data presented.

Reviewer #1: Yes

Reviewer #2: Yes

3. Has the statistical analysis been performed appropriately and rigorously?

Reviewer #1: Yes

Reviewer #2: Yes

4. Have the authors made all data underlying the findings in their manuscript fully available?

The [PLOS Data policy](http://www.plosone.org/static/policies.action#sharing) requires authors to make all data underlying the findings described in their manuscript fully available without restriction, with rare exception (please refer to the Data Availability Statement in the manuscript PDF file). The data should be provided as part of the manuscript or its supporting information or deposited to a public repository. For example, in addition to summary statistics, the data points behind means, medians and variance measures should be available. If there are restrictions on publicly sharing data—e.g. participant privacy or use of data from a third party—those must be specified.

Reviewer #1: Yes

Reviewer #2: Yes

5. Is the manuscript presented in an intelligible fashion and written in standard English?

PLOS ONE does not copyedit accepted manuscripts, so the language in submitted articles must be clear, correct, and unambiguous. Any typographical or grammatical errors should be corrected at revision, so please note any specific errors here.

Reviewer #1: Yes

Reviewer #2: Yes

**6. Review Comments to the Author**

Please use the space provided to explain your answers to the questions above. You may also include additional comments for the author, including concerns about dual publication, research ethics, or publication ethics. (Please upload your review as an attachment if it exceeds 20,000 characters)

***Reviewer #1:*** *All corrections have been addressed and well implemented. I commend the authors for this kind of a research work.

No dual publication discovered.
Ethics has been granted for this study.
Authors have adhered to publication ethics.*

**Response:** Thank you very much for this positive feedback and for agreeing to review the revised manuscript.

**Response to Reviewer 2**

***Comment 1: Methods*** *Were there any differences in findings from the study sites, that is, the clinics where participants were recruited from? Of the 87 participants, what were the distributions in numbers from each clinic (study site)? Were all interviews conducted in English?*

**Response:** We did not have sufficient statistical power to detect differences across the six clinics (< 30 participants were recruited in each clinic). Hence, we used the sub-districts as the cluster variable. The distribution of participants recruited from each site is included as a supplementary figure. All interviews were conducted in English with translations into the local languages provided where necessary.

***Comment 2: Statistical analysis*** *Line 235: Variables found to be associated with abdominal obesity in bivariate analysis (p < 0.10) and variables identified a priori in the literature were included in the multilevel models. Could you please indicate those variables that were identified a priori in the literature. This will inform the reader as to why some variables in table 4 and table 5 are included in the multilevel model when the bivariate p value is >0.10*.

**Response:** We have added the following to clarify which variables we are referring to: **“The following variables identified a priori to be associated with obesity were included in the multilevel models: multidimensional poverty (YMPI), blood pressure, family history of diabetes, sedentary behaviour, physical activity, dietary intake, household food security, and level of community violence.”**

***Comment 3: Line 240:*** *We also checked the intra-class correlation coefficient (ICC) to analyse the variability within and between sub-districts. Was there variability, this has not been discussed.*

**Response:** The ICC for the fitted models was generally low, (<0.2) indicating that there was variability between and within sub-districts. We have added this sentence to the results section. **“The ICC ranged from 0.01 to 0.2, indicating some level of variability between and within sub-districts.”**

***Comment 4: Table 1:*** *The following variables do not add up to the total (n):
Days absent from school
Dwelling type
Deceased parents*

**Response:** There were some missing values for these variables. This has been indicated in the first columns in the revised the table as follows: “Days absent from school: n= 86, Dwelling type: n= 86 and Deceased parents: n= 50.

***Comment 5: Table 2:*** *Abbreviations SSBs and MET to be written in full in the caption so that the table is self-explanatory.*

**Response:** Table 2 now includes the full descriptions for the abbreviations used in the table in the footnote placed below the table as per journal requirements. **PA= physical activity, MET= metabolic equivalent of task, SSB= sugar sweetened beverages.**

7. PLOS authors have the option to publish the peer review history of their article ([what does this mean?](https://journals.plos.org/plosone/s/editorial-and-peer-review-process#loc-peer-review-history)). If published, this will include your full peer review and any attached files.


If you choose “no”, your identity will remain anonymous but your review may still be made public.


**Do you want your identity to be public for this peer review?** For information about this choice, including consent withdrawal, please see our [Privacy Policy](https://www.plos.org/privacy-policy).

Reviewer #1: **Yes:** Prof Perpetua Modjadji

Reviewer #2: No
